# Supplementary material for: Retrograde trafficking of Argonaute 2 acts as a rate-limiting step for de novo miRNP formation on endoplasmic reticulum–attached polysomes in mammalian cells
Source: Life Sci Alliance. 2020 Feb 3;3(2):e201800161. doi: 10.26508/lsa.201800161 (PMC6998040; doi:10.26508/lsa.201800161)
Supplement: Supplementary file 1 [file LSA-2018-00161_SdataF1.pdf]

A B/Bottom **Source Data Figure 1**

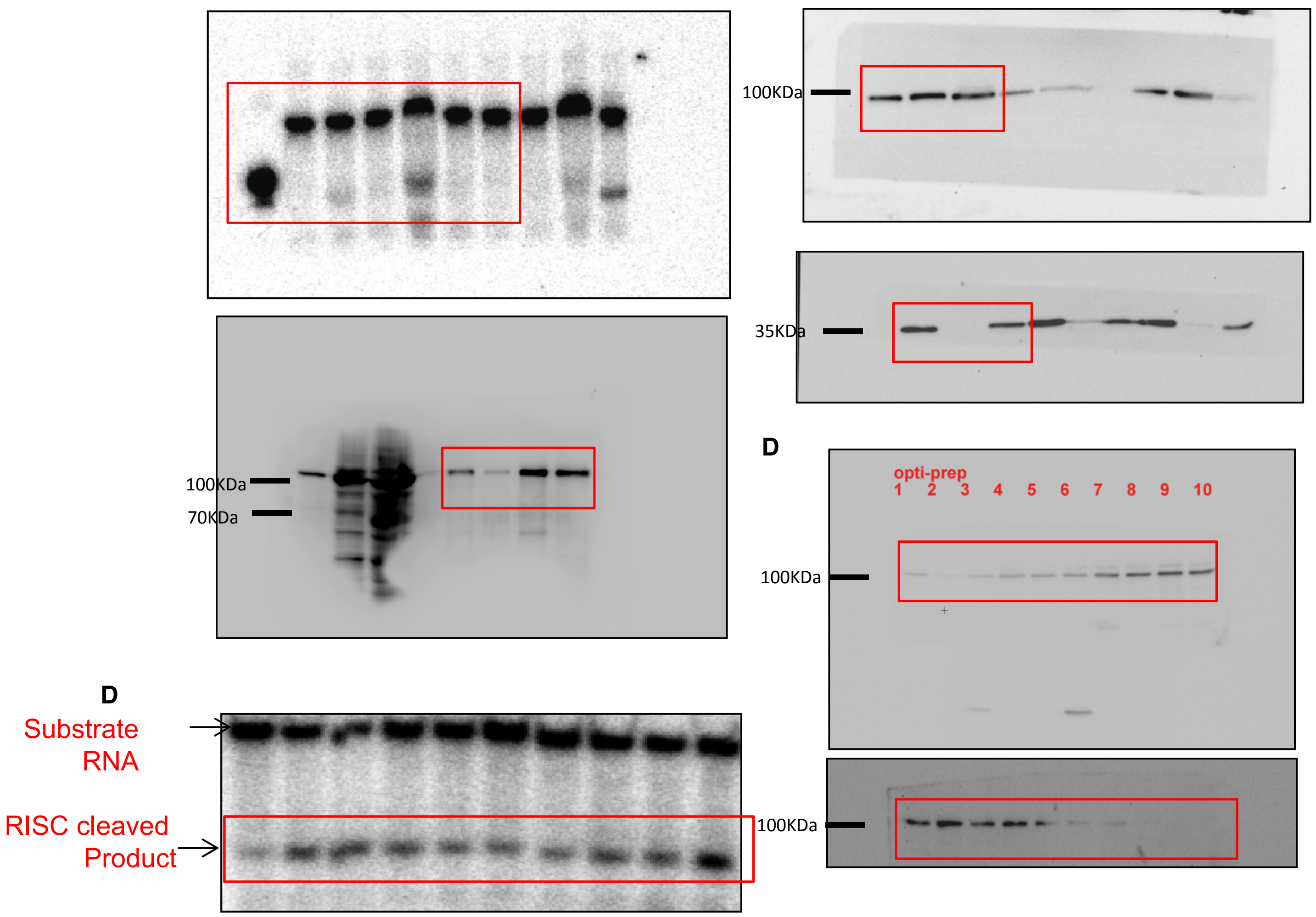

Calculation of % Specific Activity of miRISC-122				
	Densitometric Value			
Fractions	FHAGO2	RISC Cleavage	Specific Activity	**% Specific Activity**
1	83.268	0.051280412	0.000615848	6.32
2	150.116	0.116347172	0.000775048	7.95
3	168.343	0.145159103	0.000862282	8.85
4	139.78	0.107231779	0.000767147	7.87
5	93.25	0.084474857	0.000905897	9.30
6	80.29	0.075736733	0.00094329	9.68
7	73.679	0.076028442	0.001031888	10.59
8	78.053	0.104846837	0.001343277	13.78
9	81.419	0.108493241	0.00133253	13.67
10	111.672	0.130401424	0.001167718	11.98
		Total	0.009744924	

E

Target	Content	Sample	Cq	AVG. Cq	Δcq		
Let7a	Unkn	TOT	24.50	24.56066	10.97176	0	1
Let7a	Unkn	TOT	25.17				
Let7a	Unkn	TOT	24.01				
Let7a	Unkn	MIC	23.73	23.82176	9.911435	-1.06032	2.085401
Let7a	Unkn	MIC	23.77				
Let7a	Unkn	MIC	23.97				
miR 16	Unkn	TOT	20.79	20.89916	7.310256	0	1
miR 16	Unkn	TOT	20.99				
miR 16	Unkn	TOT	20.91				
miR 16	Unkn	MIC	21.29	21.254	7.343675	0.033419	0.977102
miR 16	Unkn	MIC	21.11				
miR 16	Unkn	MIC	21.36				
miR-21	Unkn	TOT	24.57	24.3782	10.7893	0	1
miR-21	Unkn	TOT	24.33				
miR-21	Unkn	TOT	24.23				
miR-21	Unkn	MIC	23.84	23.78158	9.871251	-0.91804	1.889552
miR-21	Unkn	MIC	23.80				
miR-21	Unkn	MIC	23.71				
U6 snRNA	Unkn	1	14.16	13.5889			
U6 snRNA	Unkn	1	12.71				
U6 snRNA	Unkn	1	13.02				
U6 snRNA	Unkn	2	13.56	13.91033			
U6 snRNA	Unkn	2	14.26				
U6 snRNA	Unkn	2	N/A				

Set 1

	let 7a
TOT	1
MIC	2.554624158

	miR-21
TOT	1
MIC	2.31470801

	miR-16
TOT	1
MIC	1.196953256

Sample	Target	Cq				
Total	Let7a	26.13	26.07667	5.75	0	1
Total	Let7a	26.21				
Total	Let7a	25.89				
ER	Let7a	25.01	25.62	4.36	-1.39	2.620787
ER	Let7a	25.87				
ER	Let7a	25.98				
Total	miR 16	23.12	23.09	2.763333	0	1
Total	miR 16	23.01				
Total	miR 16	23.14				
ER	miR 16	22.85	23.12	1.86	-0.90333	1.870382
ER	miR 16	22.76				
ER	miR 16	23.75				
Total	miR-21	28.45	28.62667	8.3	0	1
Total	miR-21	28.65				
Total	miR-21	28.78				
ER	miR-21	27.85	28.08667	6.826667	-1.47333	2.776627
ER	miR-21	28.14				
ER	miR-21	28.27				
Total	U6 snRNA	20.12	20.32667			
Total	U6 snRNA	20.45				
Total	U6 snRNA	20.41				
ER	U6 snRNA	21.01	21.26			
ER	U6 snRNA	21.41				
ER	U6 snRNA	21.36				

Set 3

	let 7a
TOT	1
MIC	2.620787

	miR-21
TOT	1
MIC	2.776627

	miR-16
TOT	1
MIC	1.870382

Sample	Target	Cq				
1	Let7a	25.81	25.47203	4.853987	0	1
1	Let7a	25.28				
1	Let7a	25.33				
2	Let7a	24.47	24.71172	3.364046	-1.48994	2.808774
2	Let7a	24.39				
2	Let7a	25.28				
1	miR 16	22.03	21.0339	0.41586	0	1
1	miR 16	20.70				
1	miR 16	20.37				
2	miR 16	20.47	20.63493	-0.71275	-1.12861	2.186482
2	miR 16	20.45				
2	miR 16	20.98				
1	miR-21	27.89	26.40834	5.790296	0	1
1	miR-21	26.24				
1	miR-21	25.10				
2	miR-21	25.51	25.43698	4.089304	-1.70099	3.251245
2	miR-21	25.63				
2	miR-21	25.17				
1	U6 snRNA	20.61	20.61804			
1	U6 snRNA	21.32				
1	U6 snRNA	19.93				
2	U6 snRNA	21.00	21.34768			
2	U6 snRNA	21.14				
2	U6 snRNA	21.90				

Set 2

	let 7a
TOT	1
MIC	2.808774

	miR-21
TOT	1
MIC	3.251245

	miR-16
TOT	1
MIC	2.186482

H

ER_KP EXT_RL3XB122_W_ind miR122/RL

	ER	25.20244	24.45725		
	ER	24.07429			
	ER	24.09502			
EXPI	KP SUP	26.46526	26.18614	-3.416956584	10.68086
	KP SUP	26.18184			
	KP SUP	25.91131			
	KP Pellet	30.63449	29.60309	0	1
	KP Pellet	29.23458			
	KP Pellet	28.94021			
	ER	26.5212	26.81649		
	ER	26.5573			
	ER	27.37097			
EXPII	KP SUP	25.5439	25.53981	-3.386643092	10.45878
	KP SUP	25.90466			
	KP SUP	25.17086			
	KP Pellet	28.42008	28.92645	0	1
	KP Pellet	29.2117			
	KP Pellet	29.14758			
	ER	26.12	26.26		
	ER	26.25			
	ER	26.41			
EXPIII	KP SUP	27.47	27.49333	-3.426666667	10.753
	KP SUP	27.14			
	KP SUP	27.87			
	KP Pellet	30.83	30.92	0	1
	KP Pellet	31.45			
	KP Pellet	30.48			

ER_KP EXT_RL3XB122_ W_ind miR122/miR-122	Sample	Cq			
	ER	23.96	23.69718198		
	ER	23.62			
	ER	23.51			
EXPI	KP SUP	24.72	24.73352607	-4.100346145	17.15249
	KP SUP	24.67			
	KP SUP	24.81			
	KP Pellet	28.92	28.83387222	0	1
	KP Pellet	28.84			
	KP Pellet	28.74			
	ER	25.16	25.19436219		
	ER	25.03			
	ER	25.39			
EXPII	KP SUP	26.49	24.71	-4.10247078760565	17.17777
	KP SUP	24.71			
	KP SUP	24.71			
	KP Pellet	28.52	28.81247725	0	1
	KP Pellet	28.89			
	KP Pellet	29.03			
	ER	24.15	24.28		
	ER	24.25			
	ER	24.45			
EXPIII	KP SUP	25.23	25.38	-3.94666666666666	15.41931
	KP SUP	25.45			
	KP SUP	25.47			
	KP Pellet	29.56	29.33	0	1
	KP Pellet	29.45			
	KP Pellet	28.98			
