# Supplementary material for: Retrograde trafficking of Argonaute 2 acts as a rate-limiting step for de novo miRNP formation on endoplasmic reticulum–attached polysomes in mammalian cells
Source: Life Sci Alliance. 2020 Feb 3;3(2):e201800161. doi: 10.26508/lsa.201800161 (PMC6998040; doi:10.26508/lsa.201800161)
Supplement: Supplementary file 2 [file LSA-2018-00161_SdataF2.pdf]

**Source Data Figure 2**

**B**

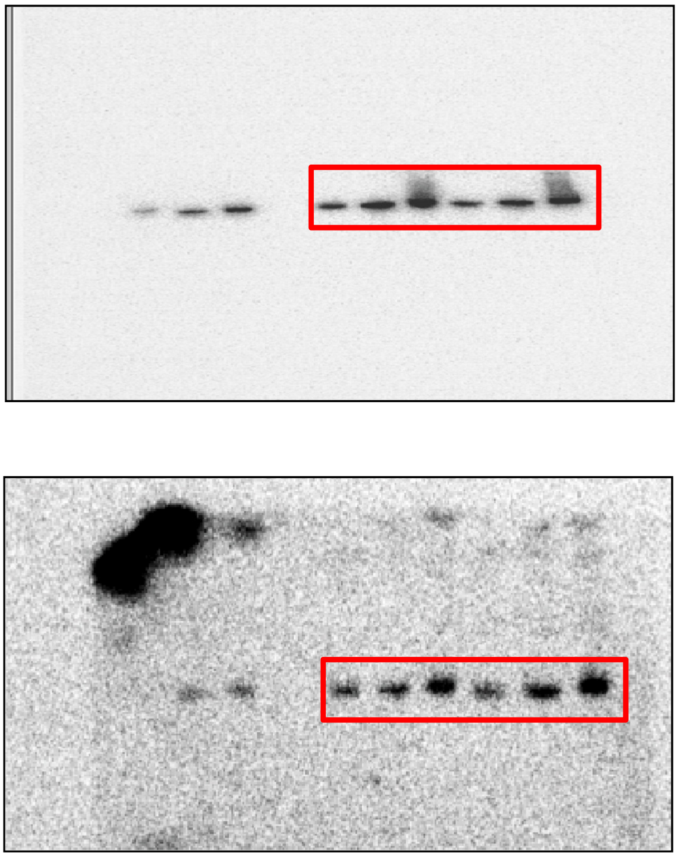

**C**

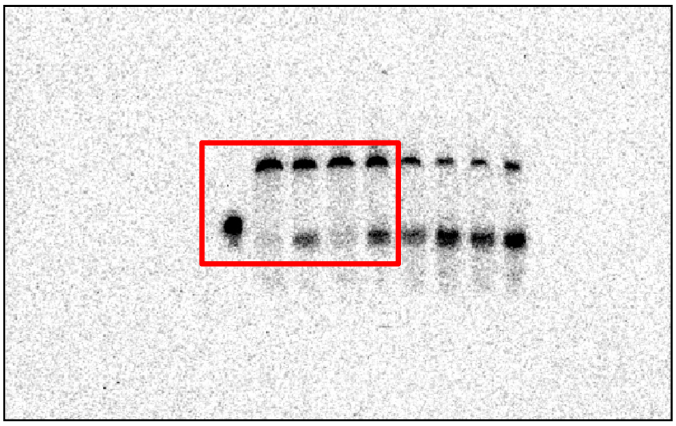

**D**

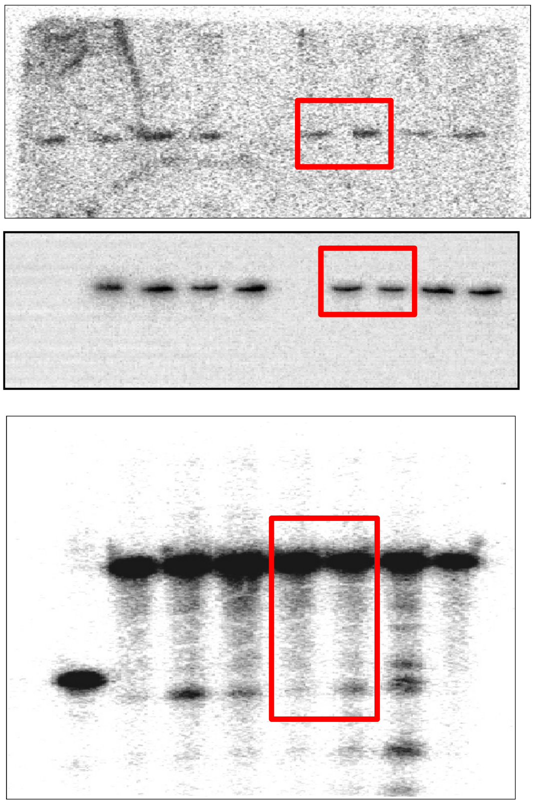

F

| | | | | | | | |
|---|---|---|---|---|---|---|---|
| EXPI | | | | | | | |
| | miR-122 | | U6 | | | | |
| with RL3xblet7 | 24 | 23.89333 | 23.58 | 23.73333 | 0.16 | 0 | 1 |
| | 23.93 | | 23.93 | | | | |
| | 23.75 | | 23.69 | | | | |
| withRL3xb122 | 21.18 | 21.35333 | 21.98 | 22.14667 | -0.79333 | -0.95333 | 1.936341 |
| | 21.36 | | 22.21 | | | | |
| | 21.52 | | 22.25 | | | | |
| EXPII | | | | | | | |
| with RL3xblet7 | 22.19 | 21.96 | 23.03 | 23.13333 | -1.17333 | 0 | 1 |
| | 21.97 | | 23.22 | | | | |
| | 21.72 | | 23.15 | | | | |
| withRL3xb122 | 20.66 | 20.86 | 23.14 | 23.15333 | -2.29333 | -1.12 | 2.17347 |
| | 20.98 | | 23.28 | | | | |
| | 20.94 | | 23.04 | | | | |
| EXPIII | | | | | | | |
| with RL3xblet7 | 23.86 | 23.805 | 23.97 | 24.10333 | -0.29833 | 0 | 1 |
| | 28.98 | | 24.11 | | | | |
| | 23.75 | | 24.23 | | | | |
| withRL3xb122 | 21.8 | 21.80667 | 22.37 | 23.18667 | -1.38 | -1.08167 | 2.11648 |
| | 21.83 | | 23.48 | | | | |
| | 21.79 | | 23.71 | | | | |

| | | | | | | | |
|---|---|---|---|---|---|---|---|
| EXPIII | miR-122 | | U6 | | | | |
| with RL3xblet7 | 25.61 | 25.35333 | 24.13 | 24.30333 | 1.05 | 0 | 1 |
| | 25.22 | | 24.34 | | | | |
| | 25.23 | | 24.44 | | | | |
| withRL3xb122 | 21.11 | 21.67 | 22.83 | 22.93 | -1.26 | -2.31 | 4.958831 |
| | 22.77 | | 22.92 | | | | |
| | 21.13 | | 23.04 | | | | |
| EXPI | | | | | | | |
| | miR-122 | | U6 | | | | |
| with RL3xblet7 | 25.01 | 25.36333 | 23.58 | 23.73333 | 1.63 | 0 | 1 |
| | 25.33 | | 23.93 | | | | |
| | 25.75 | | 23.69 | | | | |
| withRL3xb122 | 21.38 | 21.69 | 21.98 | 22.14667 | -0.45667 | -2.08667 | 4.247655 |
| | 21.36 | | 22.21 | | | | |
| | 22.33 | | 22.25 | | | | |
| EXPII | | | | | | | |
| with RL3xblet7 | 24.12 | 24.34667 | 23.03 | 23.13333 | 1.213333 | 0 | 1 |
| | 24.56 | | 23.22 | | | | |
| | 24.36 | | 23.15 | | | | |
| withRL3xb122 | 22.33 | 22.38 | 23.14 | 23.15333 | -0.77333 | -1.98667 | 3.963202 |
| | 22.54 | | 23.28 | | | | |
| | 22.27 | | 23.04 | | | | |

I

| Exp I | | | | | | |
|---|---|---|---|---|---|---|
| | | | | normalize by ago2 | | |
| Target | Cq | | | | | |
| 0h | 25.26 | 24.76023118 | 0 | 1 | 1 | 1 |
| | 24.41 | | | | | |
| | 24.61 | | | | | |
| 4h | 25.16 | 24.50 | (0.259735130943703) | 1.197258876 | 0.9 | 1.33028764 |
| | 24.12 | | | | | |
| | 24.21 | | | | | |
| 8h | 21.02 | 21.11 | (3.65141091058137) | 12.56562835 | 1.1 | 11.4232985 |
| | 20.36 | | | | | |
| | 21.95 | | | | | |
| 12h | 21.11 | 21.06507936 | -3.695151827 | 12.95243848 | 1.1 | 11.77494408 |
| | 20.77 | | | | | |
| | 21.02 | | | | | |
| 16h | 20.54 | 20.39605547 | -4.364175718 | 20.59433616 | 1.2 | 17.1619468 |
| | 20.29 | | | | | |
| | 20.36 | | | | | |
| 20h | 20.67 | 20.38365172 | -4.376579468 | 20.77216187 | 0.8 | 25.96520234 |
| | 20.34 | | | | | |
| | 20.14 | | | | | |
| 24h | 20.24 | 20.27510806 | -4.485123124 | 22.39528505 | 0.8 | 27.99410631 |
| | 20.11 | | | | | |
| | 20.47 | | | | | |

| Exp II | | | | | | |
|---|---|---|---|---|---|---|
| | | | | normalize by ago2 | | |
| Sample | Cq | | | | | |
| 0h | 30.73 | 30.34541 | 0 | 1 | 1 | 1 |
| | 30.18 | | | | | |
| | 30.13 | | | | | |
| 4h | 29.96 | 29.87 | (0.479246763121935) | 1.394015655 | 0.8 | 1.742519569 |
| | 29.85 | | | | | |
| | 29.79 | | | | | |
| 8h | 26.52 | 26.88 | (3.4613953022886) | 11.01498253 | 0.9 | 12.23886948 |
| | 26.56 | | | | | |
| | 27.56 | | | | | |
| 12h | 27.02 | 26.87835 | -3.467064285 | 11.05835039 | 0.8 | 13.82293799 |
| | 26.85 | | | | | |
| | 26.76 | | | | | |
| 16h | 26.54 | 26.40537 | -3.940044788 | 15.3487024 | 0.8 | 19.185878 |
| | 26.40 | | | | | |
| | 26.27 | | | | | |
| 20h | 26.57 | 26.31292 | -4.032497946 | 16.36450372 | 0.7 | 23.37786245 |
| | 26.32 | | | | | |
| | 26.05 | | | | | |
| 24h | 27.19 | 26.26003 | -4.08538761 | 16.97556409 | 0.6 | 28.29260682 |
| | 26.26 | | | | | |
| | 26.26 | | | | | |

| Exp III | | | | | | |
|---|---|---|---|---|---|---|
| | | | | normalize by ago2 | | |
| Sample | Cq | | | | | |
| 0h | 28.91 | 28.69992723 | 0 | 1 | 1 | 1 |
| | 28.64 | | | | | |
| | 28.55 | | | | | |
| 4h | 27.91 | 28.07 | ######### ######## | 1.551429276 | 0.9 | 1.723810307 |
| | 27.85 | | | | | |
| | 28.44 | | | | | |
| 8h | 25.02 | 25.03 | (3.6734033531325) | 12.75864623 | 0.8 | 15.94830778 |
| | 25.02 | | | | | |
| | 25.04 | | | | | |
| 12h | 25.04 | 24.92194866 | -3.777978571 | 13.71781283 | 1.1 | 12.47073894 |
| | 24.86 | | | | | |
| | 24.99 | | | | | |
| 16h | 24.54 | 24.24031795 | -4.459609282 | 22.00270939 | 1.1 | 20.00246308 |
| | 24.27 | | | | | |
| | 24.21 | | | | | |
| 20h | 24.13 | 24.12083139 | -4.579095848 | 23.90260327 | 0.9 | 26.55844808 |
| | 24.11 | | | | | |
| | 24.45 | | | | | |
| 24h | 24.35 | 24.0452124 | -4.654714831 | 25.18887582 | 0.8 | 31.48609477 |
| | 24.00 | | | | | |
| | 24.09 | | | | | |

| | | | | | normalize by ago2 | |
|---|---|---|---|---|---|---|
| Target | Cq | | | | | |
| 0h | 30.12 | 30.41146 | 0 | 1 | 1 | 1 |
| | 30.99 | | | | | |
| | 30.12 | | | | | |
| 4h | 30.22 | 30.27 | ######## | 1.10362 | 1.1 | 1.003291 |
| | 30.33 | | | | | |
| | 30.25 | | | | | |
| 8h | 29.02 | 29.44 | ######## | 1.957894 | 0.9 | 2.175438 |
| | 29.36 | | | | | |
| | 29.95 | | | | | |
| 12h | 28.11 | 29.06508 | -1.34638 | 2.542728 | 1.2 | 2.11894 |
| | 29.77 | | | | | |
| | 30.02 | | | | | |
| 16h | 29.54 | 29.39606 | -1.0154 | 2.021465 | 0.8 | 2.526831 |
| | 29.29 | | | | | |
| | 29.36 | | | | | |
| 20h | 29.67 | 29.05032 | -1.36114 | 2.568878 | 1.1 | 2.335343 |
| | 28.34 | | | | | |
| | 29.14 | | | | | |
| 24h | 29.24 | 29.27511 | -1.13635 | 2.198239 | 0.8 | 2.747799 |
| | 29.11 | | | | | |
| | 29.47 | | | | | |

| | | | | | normalize by ago2 | |
|---|---|---|---|---|---|---|
| 0h | 31.22 | 31.44378 | 0 | 1 | 1 | 1 |
| | 31.88 | | | | | |
| | 31.22 | | | | | |
| 4h | 31.21 | 31.20 | ######## | 1.187249 | 0.9 | 1.319166 |
| | 31.12 | | | | | |
| | 31.25 | | | | | |
| 8h | 30.12 | 30.13 | ######## | 2.487946 | 0.8 | 3.109933 |
| | 30.24 | | | | | |
| | 30.03 | | | | | |
| 12h | 29.56 | 29.89993 | -1.54385 | 2.915708 | 1.2 | 2.429757 |
| | 29.24 | | | | | |
| | 30.24 | | | | | |
| 16h | 30.14 | 30.20972 | -1.23405 | 2.35227 | 1.1 | 2.138427 |
| | 30.26 | | | | | |
| | 30.24 | | | | | |
| 20h | 30.57 | 30.27866 | -1.16512 | 2.242518 | 0.7 | 3.203598 |
| | 30.22 | | | | | |
| | 30.04 | | | | | |
| 24h | 30.12 | 30.33711 | -1.10667 | 2.153476 | 1.2 | 1.794564 |
| | 30.22 | | | | | |
| | 30.66 | | | | | |

| | | | | | normalize by ago2 | |
|---|---|---|---|---|---|---|
| 0h | 30.22 | 30.22221 | 0 | 1 | 1 | 1 |
| | 30.12 | | | | | |
| | 30.32 | | | | | |
| 4h | 30.01 | 30.15 | ######## | 1.052283 | 1.1 | 0.956621 |
| | 30.11 | | | | | |
| | 30.32 | | | | | |
| 8h | 29.16 | 28.85 | ######## | 2.596903 | 1.2 | 2.164086 |
| | 28.86 | | | | | |
| | 28.52 | | | | | |
| 12h | 28.11 | 29.06508 | -1.15713 | 2.230138 | 0.9 | 2.477931 |
| | 29.77 | | | | | |
| | 30.02 | | | | | |
| 16h | 28.24 | 28.70472 | -1.51749 | 2.862925 | 0.8 | 3.578657 |
| | 28.33 | | | | | |
| | 29.55 | | | | | |
| 20h | 28.55 | 28.70995 | -1.51226 | 2.852566 | 1.1 | 2.593242 |
| | 28.56 | | | | | |
| | 29.01 | | | | | |
| 24h | 29.34 | 29.18511 | -1.0371 | 2.052104 | 1.2 | 1.710087 |
| | 29.23 | | | | | |
| | 28.98 | | | | | |
