# Supplementary material for: Retrograde trafficking of Argonaute 2 acts as a rate-limiting step for de novo miRNP formation on endoplasmic reticulum–attached polysomes in mammalian cells
Source: Life Sci Alliance. 2020 Feb 3;3(2):e201800161. doi: 10.26508/lsa.201800161 (PMC6998040; doi:10.26508/lsa.201800161)
Supplement: Supplementary file 3 [file LSA-2018-00161_SdataF4.pdf]

Source Data Figure 4

A

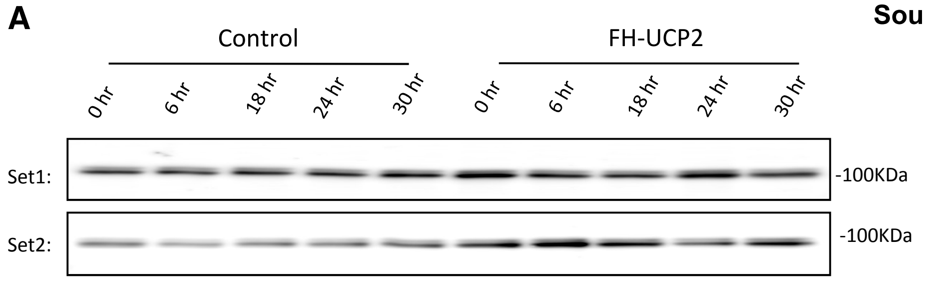

Fig. 4B Level of IP-ed AGO2

B

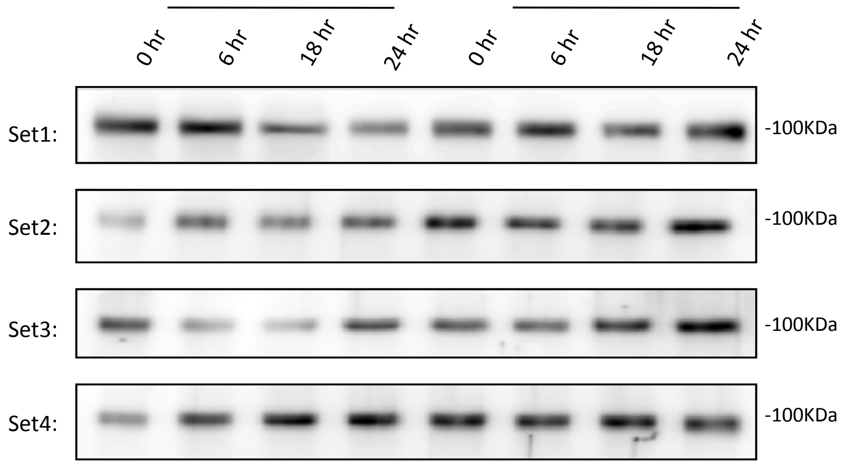

Fig. 4C Level of IP-ed AGO2

C

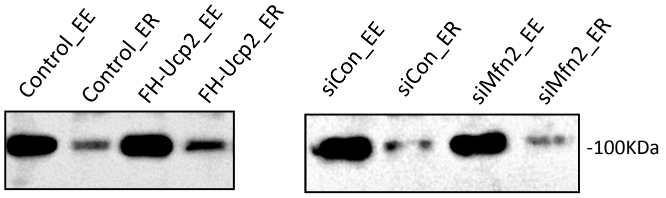

Fig. 4D Level of IP-ed Ago2

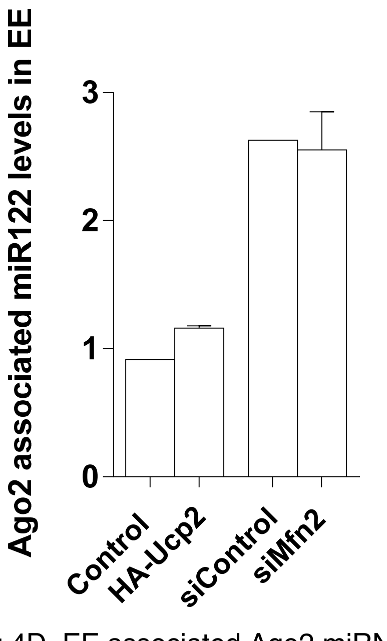

Fig 4D EE associated Ago2 miRNA bound
