# Supplementary figures and images for: Retrograde trafficking of Argonaute 2 acts as a rate-limiting step for de novo miRNP formation on endoplasmic reticulum–attached polysomes in mammalian cells
Source: Life Sci Alliance. 2020 Feb 3;3(2):e201800161. doi: 10.26508/lsa.201800161 (PMC6998040; doi:10.26508/lsa.201800161)

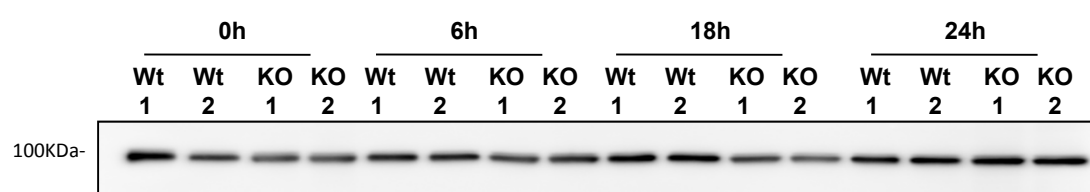

Fig. 5A Level of IP-ed AGO2

Supplement: Supplementary file 4 [file LSA-2018-00161_SdataF5.pdf]

Figure 6G

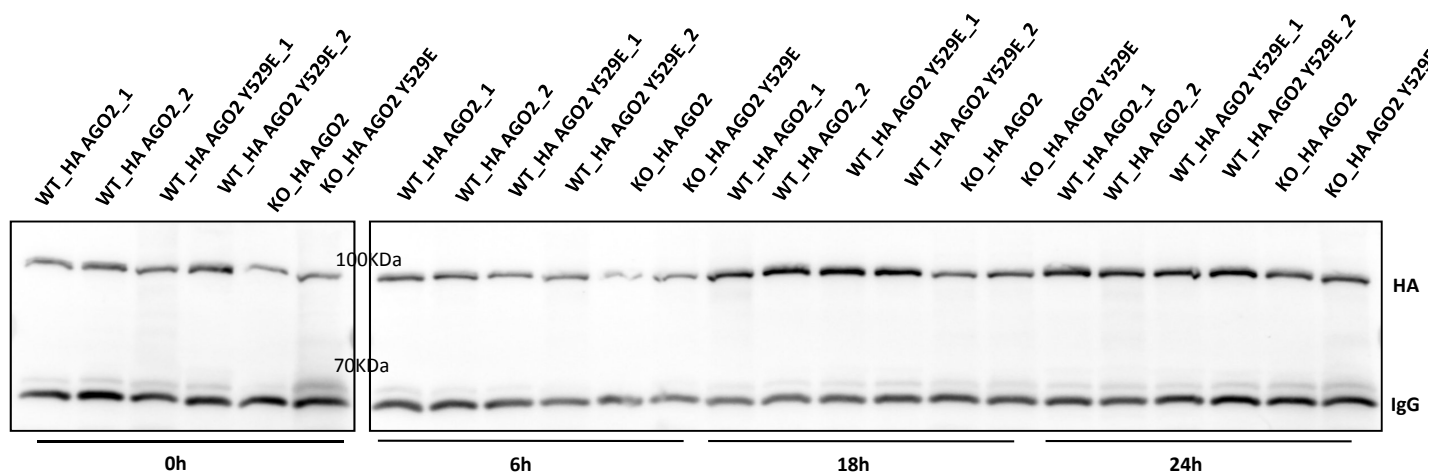

Supplement: Supplementary file 5 [file LSA-2018-00161_SdataF6.pdf]
